# Dietary Supplementation of Edible Mushroom *Phallus atrovolvatus* Aqueous Extract Attenuates Brain Changes in the *App^NL−G−F^* Mouse Model of Alzheimer’s Disease

**DOI:** 10.3390/nu17101677

**Published:** 2025-05-15

**Authors:** Raweephorn Kaewsaen, Wasaporn Preteseille Chanput, Lalida Rojanathammanee, Svetlana A. Golovko, Drew R. Seeger, Mikhail Y. Golovko, Suba Nookala, Colin K. Combs

**Affiliations:** 1 Department of Food Science and Technology, Faculty of Agro-Industry, Kasetsart University, 50 Ngam Wong Wan Rd., Ladyao, Chatuchak, Bangkok 10900, Thailand; raweephorn.k@gmail.com (R.K.); wasaporn.c@gmail.com (W.P.C.); 2 School of Sports Science, Institute of Science, Suranaree University of Technology, Nakhon Ratchasima 30000, Thailand; lalida@g.sut.ac.th; 3 Department of Biomedical Sciences, School of Medicine & Health Sciences, University of North Dakota, Grand Forks, ND 58202, USA; svetlana.golovko@und.edu (S.A.G.); drew.seeger@und.edu (D.R.S.); mikhail.golovko@und.edu (M.Y.G.); suba.nookala@und.edu (S.N.)

**Keywords:** *Phallus atrovolvatus*, mushroom, beta-glucan, Alzheimer’s disease, amyloid-beta, gliosis

## Abstract

**Background/Objectives:** Alzheimer’s disease (AD) is a neurodegenerative disease characterized by progressive dementia and brain accumulation of Aβ-peptide-containing plaques, gliosis, neuroimmune changes, and neurofibrillary tangles. Mushroom polysaccharides have been previously reported to have anti-neuroinflammation activity through the gut–brain axis. This study aimed to evaluate whether a dietary intervention with *Phallus atrovolvatus*, a recently identified edible mushroom in Thailand, could have a benefit on gut health and alleviate AD-related changes. **Methods:** Male and female 6–8-month-old littermate wild-type control (C57BL/6J) and *App^NL−G−F^* mice were randomly assigned to either a control diet or a diet supplemented with mushroom aqueous extract (MAE) for 8 weeks to quantify changes in body weight, intestine, immune cells, short chain fatty acids, brain cytokines, amyloid-β (Aβ) levels, gliosis, and memory. **Results:** MAE had no adverse effects on gut leakiness and increased pyruvate levels in serum. Splenocyte immune profiling revealed a significant increase in the frequency of IgM^+^, IA_IE^+^, and CD14^+^ cells in MAE-administered *App^NL−G−F^* female mice compared to their vehicle controls. *App^NL−G−F^* male mice that received MAE showed a significant increase in the frequency of cytotoxic CD8 T cells within the cervical lymph nodes compared to their wild-type counterparts. Aβ deposition and gliosis were significantly reduced in the hippocampi of the MAE-supplemented *App^NL−G−F^* groups. However, MAE feeding did not alter spatial recognition memory in either sex or genotype compared to their vehicle groups. **Conclusions:** Our findings demonstrated that the administration of *P. atrovolvatus* aqueous extract showed neuroprotective potential against AD-related changes in the brain with no adverse impact on gut health and memory.

## 1. Introduction

Alzheimer’s disease (AD) is a progressive neurodegenerative disorder in the elderly and causes major health problems worldwide. The common characteristics of AD patients are loss of memory and cognitive performance. Amyloid-beta (Aβ) deposition and tau protein aggregation are the main AD histopathologies, and the amyloid cascade hypothesis represents a common explanation of pathogenesis [1,2]. Briefly, the cleavage of amyloid precursor protein (APP) by the enzymes γ- and β-secretase results in the production and release of the Aβ peptide, which can oligomerize and fibrillize [3,4]. Aβ accumulation may influence neuronal cell functions and lead to synaptic loss and cell death [5,6,7]. In addition, Aβ may activate microglia and astrocytes to release neurotoxic molecules, such as pro-inflammatory cytokines, nitric oxide, and reactive oxygen species (ROS), and stimulate blood–brain barrier (BBB) dysfunction [8,9,10]. This gliosis-associated neuroinflammation may further contribute to neuronal death and cognitive impairment [11,12].

Dietary fibers are indigestible non-starch polysaccharides that can be fermented by gut microbes [13], resulting in the alteration of gut microbiota composition and the production of beneficial metabolites, such as short-chain fatty acids (SCFAs) [14]. While dietary fibers are mostly derived from the plant cell wall, mushrooms can also be a source of dietary fiber. For instance, β-glucan is a mushroom cell wall polysaccharide containing linear chains of D-glucose subunits connected by β-1,3 glycosidic bonds as a backbone and β-1,6-linked side chains [15]. Due to its structure, β-glucan is a dietary fiber that is resistant to human digestive enzymes and exhibits prebiotic potential [16]. Edible mushroom polysaccharides have been well documented for their benefits on gut health. Previous studies have demonstrated that these polysaccharides contributed positive immunoregulatory effects on intestinal health [17,18]. For instance, a polysaccharide from *Phallus indusiatus*, or bamboo mushroom, reduced pro-inflammatory cytokines, TNF-α and IL-6, and improved the gut barrier by increasing expression of tight junction proteins, Claudin-1, Occludin, ZO-1, and ZO-2, in the colon via inhibition of NF-κB and MAPK pathways in a dextran sulfate sodium-induced colitis mouse model [19]. An in vivo study conducted in ICR mice showed that hot-water extract from *Auricularia auricular* altered total SCFA content, shifted gut microbiota composition, and increased IgA and IgG levels in serum [20].

Furthermore, mushroom polysaccharides have been reported to exert anti-inflammatory activities in AD mouse models. Previous studies report that they modulate neuroinflammatory conditions through various pathways, including the inhibition of pro-inflammatory mediators, reducing Aβ accumulation, attenuating glial activation, and lowering oxidative stress [21,22,23]. For example, a polysaccharide from *Agaricus bisporus* or white button mushroom, obtained by hot water extraction, improved spatial and recognition memory assessed by a Y-maze and novel object recognition tests in D-galactose-induced aging mice. Also, it decreased proinflammatory cytokines in the brain and promoted the abundance of beneficial genera, such as *Bacteroides* and *Parabacteroides* [24]. *Sparassis crispa* polysaccharide showed neuroprotective effects in a D-galactose-and-AlCl_3_-induced AD mouse model. The supplementation of *S. crispa* for 4 weeks ameliorated learning deficits, amyloidogenesis, and glial activation and modulated γ-aminobutyric acid, glutamate, and acetylcholine levels in the brain [25]. Since these prior studies have indicated that edible mushrooms alleviate neuroinflammatory changes, further investigation of new mushrooms and their polysaccharides could increase knowledge of their positive health effects.

*Phallus atrovolvatus* (Syn. *Dictyophora duplicata* (Bosc.) E. Fisch.) is an edible mushroom that was recently discovered in Thailand. This mushroom is scientifically close to a bamboo mushroom, *P. indusiatus*, which is widely consumed in China and other Asian countries due to its taste, nutritional value, and various health benefits [26]. Currently, this wild edible mushroom is identified as a Thai strain of bamboo mushroom and is commercially cultivated in many parts of Thailand with increasing economic potential. Crude polysaccharides of *P. atrovolvatus* have been previously reported to have anti-oxidative properties [27] and gut immunomodulatory activity in a colitis mouse model [28]. Our prior work demonstrated that the *P. atrovolvatus* mushroom, in both cooked mushroom and aqueous extract forms, exerted gut health benefits through the promotion of SCFA production in correlation to its β-glucan content and modulation of gut microbiota population. It also promoted the growth of the probiotic genera *Bacteroides*, *Bifidobacterium*, *Clostridium sensu stricto 1*, and *Streptococcus* and suppressed pathogenic bacterial groups, *Escherichia-Shigella*, *Klebsiella*, and *Veillonella* [29].

Therefore, in this study, we aimed to explore a new health benefit of *P. atrovolvatus* mushroom focused on brain neuroinflammatory changes and potential signaling molecules using a mouse model of AD. The mushroom aqueous extract was administered to wild-type and *App^NL−G−F^* mice for 8 weeks to examine the impacts on neuroimmune changes, AD-type pathology, and memory performance. Our outcomes provide a new biological function of this mushroom and its potential application as a novel dietary supplementation for AD treatment.

## 2. Materials and Methods

### 2.1. Preparation of Mushroom Aqueous Extract (MAE)

The raw mushroom material was washed, dried, and powdered. The MAE was prepared by triply repeated hot-water extraction coupled with α-amylase treatment as outlined in our previous study [29]. The obtained crude polysaccharide sample was further treated with an alkaline-ethanol treatment to remove the lipopolysaccharide (LPS) present in the sample. LPS inactivation was performed according to a method described by a previous study with a slight modification [30]. A total of 500 mg of the sample was mixed with 100 mL of 1 M NaOH in 60% ethanol and incubated at 56 °C for 5 h while stirring. The mixture was neutralized with 1 M HCl and brought to 400 mL with absolute ethanol and kept at 4 °C overnight. After that, the mixture was centrifuged at 7000× *g* for 20 min, washed three times with ethanol, and centrifuged again to recover the gel-like pellet. The sample was dried, powdered, and kept at −20 °C for further analysis. The sample after LPS inactivation was quantified for LPS content using a ToxinSensor^TM^ Chromogenic LAL Endotoxin Assay Kit (Piscataway, NJ, USA) according to the manufacturer’s protocol. LPS concentration was calculated based on a standard curve of *Escherichia coli* endotoxin standard. In this study, the LPS-inactivated sample was called “Mushroom aqueous extract (MAE)” and subjected to chemical composition analysis and animal study.

### 2.2. Chemical Composition Analysis of MAE

Total protein content was determined using the Kjeldahl method outlined by AOAC 1995 [31]. The amounts of total glucan and α- and β-glucan were quantified using a β-Glucan Assay Kit (Yeast & Mushroom, K-YBGL; Megazyme; Bray, Ireland) according to the manufacturer’s protocol.

### 2.3. Animal Models

Wild-type (WT) C57BL/6J mice were originally purchased from Jackson Laboratories and were generated as littermate controls of the *App^NL−G−F^* mice. The *App^NL−G−F^* mice were originally obtained from RIKEN Center for Brain Science, Japan, and are maintained as a colony at the University of North Dakota. With three mutations of humanized Aβ regions, including Swedish (NL), Arctic (G), and Iberian (F), these transgenic mice develop familial human AD pathology without the overexpression of APP [32]. Mice at 6–8 months of age (*n* = 9–11 per treatment group) were used in this study. All protocols of animal use were approved by the Institutional Animal Care and Use Committee (IACUC) at the University of North Dakota. The animals had access to food and water ad libitum and were maintained under standard housing conditions (12 h light/dark cycle and 22 ± 1 °C).

### 2.4. Treatments

All mice were randomly divided into 8 experimental groups according to their genotype, sex, and treatment; (1) male/WT/vehicle, (2) male/WT/MAE, (3) male/*App^NL−G−F^*/vehicle, (4) male/*App^NL−G−F^*/MAE, (5) female/WT/vehicle, (6) female/WT/MAE, (7) female/*App^NL−G−F^*/vehicle, and (8) female/*App^NL−G−F^*/MAE. In this study, MediGel^®^Sucralose (Clear H_2_O, Portland, ME, USA), which is a flavored and soft water gel containing 99% purified water, was used as a vehicle and water substitute. MAE was suspended in the vehicle at a concentration of 0.5 mg MAE/g vehicle and administered to animals with an estimated mean water consumption of 5 mL or g/25 g mouse/day, to reach a dose of MAE of 100 mg/kg body weight (BW). All mice were provided with either vehicle or vehicle supplemented with MAE for 8 weeks ad libitum. The amounts of consumed vehicle, MAE, and mouse body weights were followed every other day throughout the treatment.

### 2.5. Tissue Collection

At the end of the feeding period, mice were euthanized, followed by cardiac perfusion using ice-cold sterile Ca^2+^ and Mg^2+^-free phosphate-buffered saline (PBS). Cervical lymph nodes, spleens, and blood were collected for immune cell phenotyping analysis. Blood, intestines, and brains were collected for biochemical analysis. The right hemispheres of the brains were separated into hippocampus and cortical regions, while the left hemisphere of each brain was fixed in 4% paraformaldehyde for immunohistochemistry. All tissues were flash-frozen in liquid nitrogen and kept at −80 °C.

### 2.6. Enzyme-Linked Immunosorbent Assay (ELISA)

LPS levels. The blood samples were centrifuged at 2000× *g* for 10 min to collect serum. LPS concentrations were determined by a lipopolysaccharide ELISA kit (MyBioSource, San Diego, CA, USA) according to the manufacturer’s protocol.

Lipocalin and tight junction protein (ZO-1) levels. A mid-portion of the flash-frozen colons was weighed and lysed in ice-cold RIPA buffer (20 mM Tris-HCl, pH 7.4, 150 mM NaCl, 1 mM Na_3_VO_4_, 10 mM NaF, 1 mM EDTA, 1 mM EGTA, 0.2 mM phenylmethylsulfonyl fluoride, 1% Triton-X100, 0.1% SDS, and 0.5% deoxycholate) with protease inhibitors (1 mM AEBSF, 0.8 μM aprotinin, 21 μM leupeptin, 36 μM bestatin, 15 μM pepstatin A, 14 μM E-64). The tissue samples were homogenized using Zirconium oxide beads (MidSci, St. Louis, MO, USA) in a bullet blender (Quasar instruments, Colorado Springs, CO, USA) and centrifuged (20,000× *g*, 4 °C, 10 min). The supernatant was collected, and protein concentrations were quantified by the bicinchoninic acid (BCA) assay (Thermo Fisher Scientific, Waltham, MA, USA). The Quantikine^®^ ELISA Mouse Lipocalin-2/NGAL Immunoassay (R&D systems, Minneapolis, MN, USA) Kit and Mouse Tight Junction Protein ZO-1 (TJP1) ELISA Kit (MyBioSource, San Diego, CA, USA) were used to quantify the levels of lipocalin and tight junction protein, ZO-1, from the colon lysates, respectively.

Soluble and insoluble Aβ 1-40 and Aβ 1-42 levels. Each flash-frozen hippocampus was weighed and lysed in ice-cold RIPA buffer with protease inhibitors. The tissue samples were homogenized using zirconium oxide beads in a bullet blender and centrifuged. The supernatant was collected as the “soluble fraction”. The resulting pellet was resuspended in a 5 M guanidine HCl/50 mM Tris-HCl, pH 8.0 solution. The mixture was then homogenized and centrifuged again. The collected supernatant was used as the “insoluble fraction”. All lysate samples were measured for protein concentration by the BCA assay. All fractions were used to determine the levels of Aβ 1-40 and Aβ 1-42 using the Quantikine™ ELISA Human Amyloid β (aa1-40) Immunoassay Kit and the Quantikine™ ELISA Human Amyloid β (aa1-42) Immunoassay Kit (R&D systems, Minneapolis, MN, USA), respectively [33].

### 2.7. Phenotyping of Immune Subsets by Flow Cytometry

After collecting cervical lymph nodes and spleens in Ca^2+^ and Mg^2+^-free PBS, cell dissociation was performed by mashing the tissues with a plunger of a syringe over a 40 µm cell strainer. Cells were washed with ice-cold PBS and centrifuged to obtain cell pellets. The resulting cell pellets and whole blood samples were resuspended in FACS buffer (Ca^2+^ and Mg^2+^-free PBS with 2% fetal bovine serum). RBC lysis buffer (eBioscience, ThermoFisher Scientific, Waltham, CA, USA) was then used to lyse red blood cells. Afterward, cells were resuspended in 150 μL of Live/Dead (Ghost dye 510, 1:1000; Tonbo BioSciences, San Diego, CA, USA) dye prepared in Ca^2+^ and Mg^2+^-free PBS. The cells were incubated at 4 °C for 30 min, spun down, and washed with FACS buffer. The cells were resuspended in 150 μL of Fc Block (purified rat anti-mouse CD16/CD32, clone 2.4G2 (Biolegend, San Diego, CA, USA) prepared in 1:50 dilution in FACS buffer, followed by incubation on ice for 10 min. The cells were then stained with an antibody cocktail including APC-Cy7-labeled CD45 (Biolegend), BV605-labeled CD4 (Biolegend), BV785-labeled CD8a (Biolegend), PE-Cy7-labeled CD62L (Biolegend), Alexa fluor700 700-labeled Ki-67 (Biolegend), PE-labeled FOXP3 (eBioscience), PE-Cy5-labeled IgM (Biolegend), APC-Cy7-labeled MHC-II (IA_IE) (Biolegend), and BV605-labeled CD14 (Biolegend). At least 10,000 events were collected on a flow cytometer (BD FACS symphony A3). Acquired data were analyzed using FlowJo^TM^ v10.8.1 software (BD Life Sciences, Franklin Lakes, NJ, USA) [33].

### 2.8. Short Chain Fatty Acid Analysis

A piece of the flash-frozen parietal cortex was weighed and sonicated in 300 μL of methanol containing butyrate-^13^C_4_, and lactate-^13^C_3_ (20 ng each) as internal standards. A 10 μL measure of serum was brought up to 300 μL with methanol containing internal standards. After centrifugation at 20,000× *g*, 4 °C, 10 min, the supernatants from all samples were subjected to derivatization and SCFA analysis as described in a previous study [34]. The carboxylic acid moiety of SCFAs was analyzed as nitrophenyl hydrazide derivatives (CANPD). All reagents for derivatization were of mass spectrometry grade. In brief, a 250 μL sample was brought up to 300 μL with methanol and mixed with 300 μL of pyridine, 300 μL of 1-ethyl-3-(3-dimethylaminopropyl) carbodiimide (250 mM in methanol), and 300 μL of 2-Nitrophenyl hydrazine hydrochloride (20 mM in methanol). The mixture was incubated at 60 °C for 20 min in a water bath, followed by the addition of 200 μL of potassium hydroxide (15% in 20% methanol), and incubated at 60 °C for another 20 min. Next, the mixture was added to 3 mL of phosphoric acid (0.5 mM in water) and 4 mL of ether to extract CANPD. The extract was washed twice with 4 mL of water by centrifugation at 1500× *g* for 5 min. A top layer was collected as CANPD extract, evaporated under a nitrogen stream, and then transferred into a silanized microvial insert (part#5181–8872; Agilent, Santa Clara, CA, USA). The CANPD was mixed with 100 μL of ether, evaporated, and re-dissolved in 20 μL of 50% methanol in water. Ten microliters of each sample was subjected to UPLC-MS/MS (Waters, Milford, MA, USA). CANPD was resolved using Waters ACUITY UPLC HSS T3 column (1.8 μM, 100 Å pore diameter, 2.1 × 150 mm, Waters, Milford, MA, USA) using a gradient of water with 0.1% formic acid and acetonitrile with 0.1% formic [34]. For detection, Waters Q-TOF Synapt G2X with an electrospray ionization ion source was operated in negative ion mode, and data acquisition was performed in MS^e^ mode. Nitrophenyl hydrazide derivatives of acetate (*m*/*z* = 194.0566), propionate (*m*/*z* = 208.0722), and butyrate and isobutyrate (*m*/*z* = 222.0879) were quantified against butyrate-^13^C_4_ (*m*/*z* = 226.1013). Lactate (*m*/*z* = 224.0671) and pyruvate (*m*/*z* = 222.0515) were quantified against lactate-^13^C_3_ (*m*/*z* = 227.0772).

### 2.9. Th1/Th2/Th17 Cytokine Measurement

Temporal cortex lysates were used for cytokine analysis. First, each temporal cortex was weighed and lysed in RIPA buffer with protease inhibitors. The tissue samples were homogenized using zirconium oxide beads in a bullet blender and centrifuged (5000× *g*, 4 °C, 10 min). The supernatant was collected, and protein content was estimated by the BCA assay. All samples were loaded at the same protein concentration for cytokine assessment. RayBiotech Quantibody^®^ Mouse Th1/Th2/Th17 Q1 arrays (QAM-TH17-1-1, Ray-Biotech, Norcross, GA, USA) were used to determine brain cytokine levels according to the manufacturer’s protocol. Eighteen cytokines, including IL-1β, IL-2, IL-4, IL-5, IL-6, IL-10, IL-12p70, IL-13, IL-17, IL-17F, IL-21, IL-22, IL-23, IL28, IFN-γ, MIP-3α, TGF-β, and TNF-α, were evaluated. The arrays were sent to RayBiotech and scanned using a GenePix 4400 scanner for scanning the slide arrays. The cytokine levels (pg/mL) were calculated based on a standard curve of each cytokine using the RayBiotech analysis tool.

### 2.10. Immunohistochemistry

After tissue collection, the fixed left-brain hemisphere of each mouse was histologically processed according to prior work [35]. First, the fixed brain samples were cryoprotected in a gradient of 15% sucrose-PBS solution for 72 h, followed by a 30% sucrose–PBS solution for another 72 h. Next, the brain tissues were embedded in a 15% gelatin–PBS matrix and submerged in 4% paraformaldehyde overnight. For cryoprotection, the prepared gelatin–brain blocks were changed twice in 30% sucrose–PBS solution for 72 h. Each block was flash-frozen by submerging it in dry ice/isopentane and was serially cut into 40 μm sections using a Leica 2010R sliding microtome with a freezing stage (Physitemp, Clifton, NJ, USA). The tissue sections were stored in PBS containing 0.1% sodium azide at 4 °C until immunostaining. The brain sections from all treatment groups were subsequently stained using antibodies against Aβ (1:500 dilution, rabbit, D54D2; Cell Signaling Technology, Danvers, MA, USA), Iba-1 (1:1000 dilution, rabbit, 019-19741; Wako Chemicals USA, Richmond, VA, USA), and GFAP (1:1000 dilution, rabbit, D1F4Q; Cell Signaling Technology, Danvers, MA, USA) to detect Aβ deposition, microglia, and astrocytes, respectively, as previously described [33]. Antigen retrieval was performed for Aβ and Iba-1 before staining. For Aβ antigen retrieval, the brain sections were incubated in 25% formic acid for 25 min at room temperature, then washed with distilled water and PBS. For Iba-1, the brain sections were incubated in Tris-EDTA solution (10 mM Tris-base, 1 mM EDTA, pH 9) at 95 °C for 10 min, then washed with distilled water and PBS before blocking. After antigen retrieval, the brain sections were subsequently incubated in 0.3% H_2_O_2_ for 5 min, then blocked in PBS containing 5% normal goat serum (Equitech-Bio, Kerrville, TX, USA), 0.5% bovine serum albumin (Equitech-Bio, Kerrville, TX, USA), 0.1% Triton X-100 (Sigma-Aldrich, St. Louis, MO, USA), and 0.02% sodium azide for at least 1 h. Afterward, the sections were incubated in the primary antibody at 4 °C overnight, washed, and incubated in a biotinylated secondary antibody (1:2000) for 2 h at room temperature. Next, the sections were further incubated using a VECTASTAIN Avidin/biotin Complex (ABC) Kit and a Vector VIP Peroxidase (HRP) Substrate Kit (SK-4600; Vector Laboratories, Newark, CA, USA) to visualize antibody binding. The immunostained sections were mounted onto gelatin-subbed slides, dehydrated, and cover-slipped using Permount (Fisher Scientific, Portsmouth, NH, USA). The brain slides were imaged using a Hamamatsu NanoZoomer 2.0-HT Brightfield Scanning system. Immunoreactivity quantification was performed using an open-source digitalized image analysis platform, QuPath (v.0.4.3) [36]. The hippocampus regions of all brain sections were annotated. For staining quantification, QuPath software grouped adjacent and similar pixels into a superpixel of 25 mm^2^ and applied an arbitrary intensity value for each superpixel to identify a positive or negative value [37]. The results were calculated by QuPath and expressed as % positive superpixels.

### 2.11. Behavior Test

The cross-maze test was performed to assess the spatial working memory of each animal at the end of the 8-week treatment. The procedure followed our previous protocol [38]. The maze was arranged in a cross-configuration, consisting of 4 arms, 30 cm wide each, and a central 5 cm^2^ area. Each arm had a 15 cm high wall along each side and at the end, and the central area of the maze was open. Each mouse was placed gently at the end of one arm as a starting point and allowed to explore the maze freely for 10 min. The order of arm choices was recorded and analyzed using ANY-maze software version 7.20 (Stoelting Co., Wood Dale, IL, USA). A correct alternation was defined as visiting each of the 4 arms without re-visiting any arms. The number of alternations was counted, and the percentage of alternation was calculated as follows: Number of alterations/(Number of total entitries-3).

### 2.12. Statistical Analysis

Results are expressed as mean values ± SEM. Outliers were tested by the ROUT method (Q = 1) and removed for each data set before plotting and the final n is indicated in each figure legend. Statistical analysis was performed by two-way ANOVA followed by Tukey’s multiple comparisons tests using GraphPad Prism 10.3.1 software (GraphPad Software, San Diego, CA, USA). Significance is indicated by *p*-value measurement with *p* < 0.05 considered statistically significant; * *p* < 0.05; ** *p* < 0.01; *** *p* < 0.001; **** *p* < 0.0001.

## 3. Results

### 3.1. Chemical Composition of MAE

MAE was determined for glucan, protein, and LPS contamination content. Glucans were a major component, which represented 46.30 ± 0.58% of the total weight. The amounts of α- and β-glucan were 16.23 ± 0.23% and 30.06 ± 0.81%, respectively. Total protein content was 13.31 ± 0.02%. The LPS present in the MAE was 0.028 ± 0.007%.

### 3.2. Consumption of Vehicle and Dose Received of MAE and β-Glucan

Amounts of consumed MediGel^®^Sucralose supplemented with and without MAE (vehicle +/− MAE) were followed throughout the experimental period. Weights of vehicle +/− MAE before and after feeding to the animals were recorded every two days and the amount of consumed vehicle +/− MAE was calculated for the obtained dose. As shown in Table 1, the consumption of vehicle +/− MAE was in the range of 6.71–8.17 g/mouse/day, resulting in obtained doses of MAE in the MAE-treated groups ranging from 132.0–143.1 mg/kg BW/mouse/day. As β-glucan is the major glucan component and is known as a dietary fiber, the doses of β-glucan were also calculated. The results indicated that the amounts of received β-glucan were 39.61–42.93 mg/kg BW/mouse/day (Table 1).

### 3.3. Body Weight Trend

**Figure 1:**
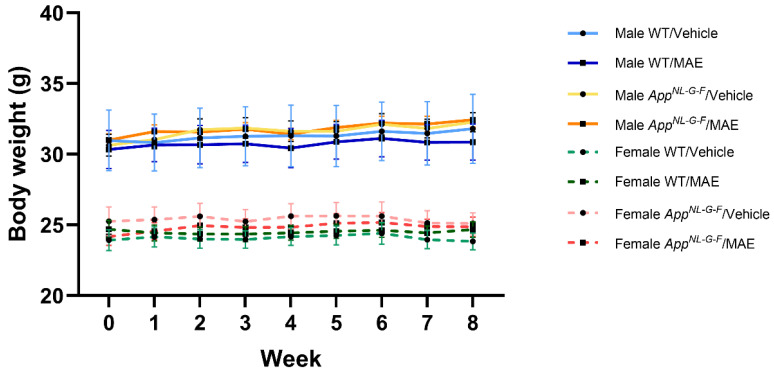
Body weights of all wild-type (WT) and *App^NL−G−F^* mice with and without MAE supplementation. The data shown are mean ± SEM (*n* = 9–11 per group).

On the initial day, the weights of male and female mice were approximately 30 and 25 g, respectively, with male mice having a normally larger body size and weight than females (Figure 1). At the end of the treatment, the body weights of all animal groups were not significantly different (*p* ≥ 0.05) (Figure 1).

### 3.4. Effects of MAE Supplementation on Gut Inflammation and Serum LPS Levels

**Figure 2:**
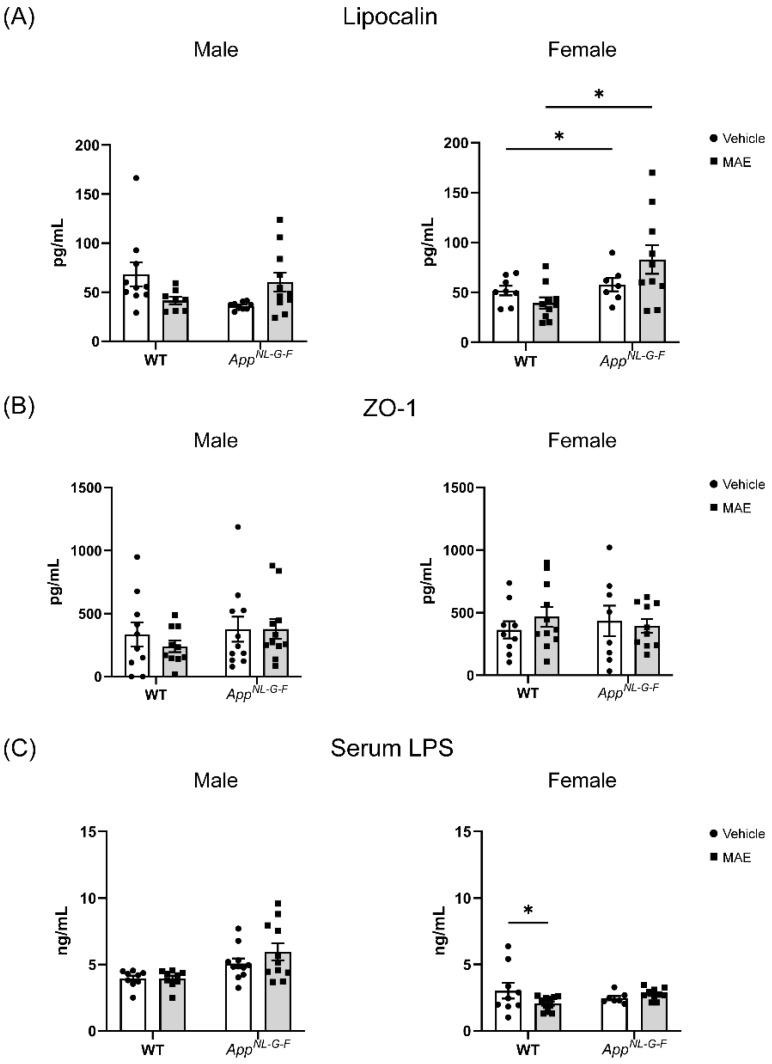
Effects of mushroom aqueous extract (MAE) supplementation on (**A**) levels of lipocalin, (**B**) tight junction protein (ZO-1) in colons, and (**C**) LPS in blood serum of wild-type (WT) and *App^NL−G−F^* mice. Values are expressed as the mean ± SEM (*n* = 7–11 per group). Two-way ANOVA followed by Tukey’s test was performed to determine statistical differences; * *p* < 0.05.

Lipocalin and tight junction protein (ZO-1) levels were determined to evaluate the effects of MAE supplementation on gut health. As shown in Figure 2A, there was no significant change (*p* ≥ 0.05) in lipocalin levels in all male groups. In female groups, *App^NL−G−F^* mice had significantly higher (*p* < 0.05) lipocalin levels than WT mice. ZO-1 level was measured in colon lysates to determine the effect of MAE treatment on intestinal barrier integrity (Figure 2B). According to the results, no significant change (*p* ≥ 0.05) in ZO-1 levels was observed in any animal groups after 8 weeks of treatment. In addition, a significant reduction (*p* < 0.05) in the serum LPS level was found in female WT mice treated with MAE, compared to their vehicle group, while there was no significant change (*p* ≥ 0.05) observed in other animal groups (Figure 2C).

### 3.5. Effects of MAE Supplementation on Modulation of Immune Cells

**Figure 3:**
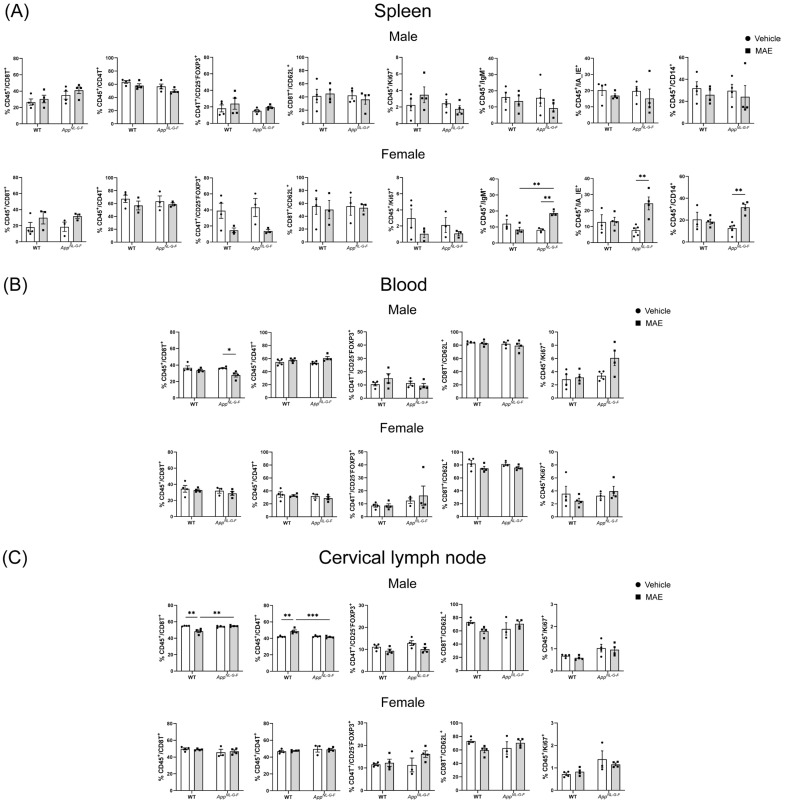
Effects of mushroom aqueous extract (MAE) supplementation on changes in immune cells in wild-type (WT) and *App^NL−G−F^* mice after the 8-week treatment. (**A**) Spleen, (**B**) blood, and (**C**) cervical lymph nodes were collected for flow cytometric analysis of surface markers. Each dot represents a single mouse, and bars indicate mean values of % cells gated through live cells. Values are expressed as mean ± SEM (*n* = 3–5 per group). Two-way ANOVA followed by Tukey’s test was performed to determine statistical differences; * *p* < 0.05, ** *p* < 0.01, and *** *p* < 0.001.

Immune cell populations in spleens, blood, and cervical lymph nodes were determined through flow cytometry to assess the impact of MAE treatment on immune responses. As shown in Figure 3, numbers of CD8^+^ (cytotoxic T cells), CD4^+^ (helper T cells), FOXP3^+^ (regulatory T cells), CD62L^+^ (Naïve T cells), Ki67^+^ (cell proliferation marker), IgM^+^ (B cells), IA_IE^+^ (antigen-presenting cells), and CD14^+^ (monocytes and macrophages) were identified.

In Figure 3A, our results showed that the modulation of immune cells in spleens due to MAE supplementation was observed in female groups. The levels of IgM^+^, IA_IE^+^, and CD14^+^ were significantly increased (*p* < 0.05) in female MAE-treated *App^NL−G−F^* mice, while there was no significant change in male groups. For blood, a significant reduction (*p* < 0.05) in CD8^+^ cells was only observed in male *App^NL−G−F^* mice supplemented with MAE compared to their vehicle group, while there were no significant differences (*p* ≥ 0.05) in the other groups (Figure 3B). The quantification of immune cells in cervical lymph nodes (Figure 3C) showed that MAE treatment significantly reduced (*p* < 0.05) the frequency of CD8^+^ cells in male WT groups. In addition, the frequency of CD8^+^ cells in MAE-treated *App^NL−G−F^* mice was significantly higher (*p* < 0.05) than in MAE-treated WT mice. The percentage of CD4^+^ cells in the male MAE-fed WT group was significantly higher (*p* < 0.05) than their vehicle control. Male MAE-treated *App^NL−G−F^* mice showed a significantly lower (*p* < 0.05) level of CD4^+^ cells than MAE-treated WT mice.

### 3.6. Effects of MAE Supplementation on SCFA Production

**Figure 4:**
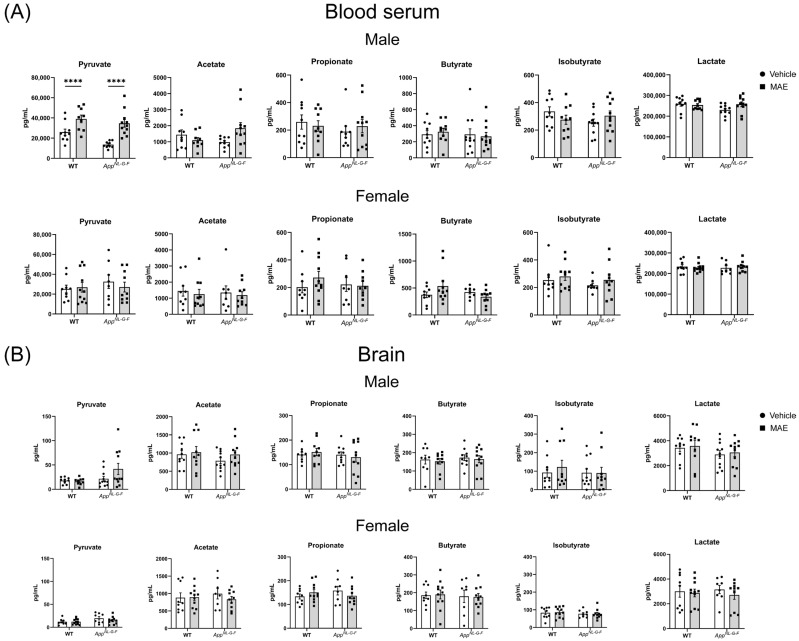
Effects of mushroom aqueous extract (MAE) supplementation on concentrations of SCFAs in (**A**) blood serum and (**B**) brain parietal cortices of wild-type (WT) and *App^NL−G−F^* mice after 8 weeks of treatment. Pyruvate, acetate, propionate, butyrate, isobutyrate, and lactate were measured using mass spectrometry. Values are expressed as mean ± SEM (*n* = 8–11 per group). Two-way ANOVA followed by Tukey’s test was performed to determine statistical differences; **** *p* < 0.0001.

To investigate the impact of MAE supplementation on changes in SCFAs, levels of pyruvate, acetate, propionate, butyrate, isobutyrate, and lactate were measured to observe SCFA concentrations in serum and parietal cortex of the brain. As shown in Figure 4A, MAE-supplemented groups of male WT and *App^NL−G−F^* mice had significantly higher (*p* < 0.05) levels of pyruvate in serum than their respective vehicle groups, while there was no significant change (*p* ≥ 0.05) of other SCFAs levels in the other groups. No significant changes (*p* ≥ 0.05) were observed in the brains of all animal groups (Figure 4B).

### 3.7. Effects of MAE Supplementation on Brain Cytokines

**Figure 5:**
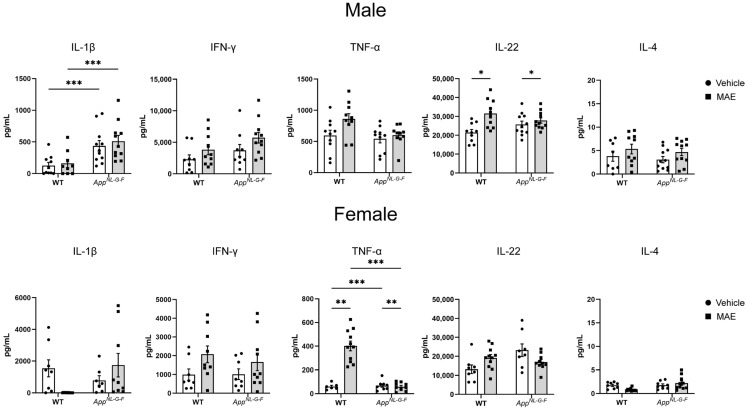
Effects of mushroom aqueous extract (MAE) supplementation on brain temporal cortex cytokine levels of wild-type (WT) and *App^NL−G−F^* mice after 8 weeks of treatment. Values are expressed as mean ± SEM (*n* = 6–11 per group). Two-way ANOVA followed by Tukey’s test was performed to determine statistical differences; * *p* < 0.05, ** *p* < 0.01, and *** *p* < 0.001.

Brain cytokines were measured in lysates from temporal cortices of WT and *App^NL−G−F^* mice (Figure 5). In male groups, *App^NL−G−F^* mice had significantly higher (*p* < 0.05) levels of IL-1β compared to WT mice. MAE supplementation significantly upregulated (*p* < 0.05) levels of IL-22 in both WT and *App^NL−G−F^* groups, while there were no effects on the other cytokines. For female mice, in the vehicle groups, the levels of TNF-α were observed to be significantly higher (*p* < 0.05) in *App^NL−G−F^* mice than in WT mice. Compared to the vehicle groups, MAE treatment significantly increased (*p* < 0.05) the levels of TNF-α in female WT mice, and a significant reduction (*p* < 0.05) in TNF-α levels was observed in female *App^NL−G−F^* mice.

### 3.8. Effects of MAE Supplementation on Hippocampal Aβ Deposition

**Figure 6:**
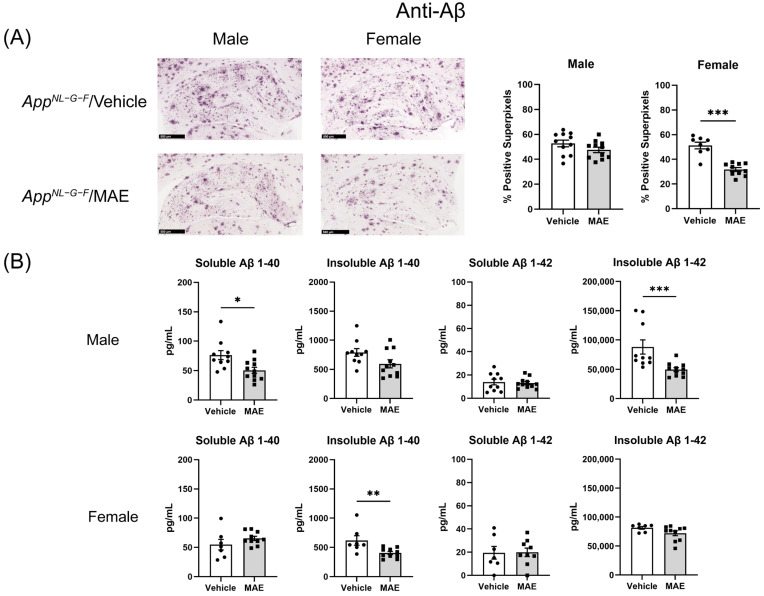
Effects of mushroom aqueous extract (MAE) supplementation on alteration of Aβ accumulation in the hippocampus of *App^NL−G−F^* mice after 8-week treatment. (**A**) Brain sections of each animal were stained using anti-Aβ immunohistochemistry. Scale bar, 500 μm. The percentages of Aβ-positive superpixels in the hippocampus were determined from at least three sections per mouse in each treatment group. (**B**) Levels of soluble and insoluble Aβ 1-40 and 1-42 in the hippocampal lysates of *App^NL−G−F^* mice were measured by ELISA. Values are expressed as mean ± SEM (*n* = 7–11 per group). Two-way ANOVA followed by Tukey’s test was performed to determine statistical differences; * *p* < 0.05, ** *p* < 0.01, and *** *p* < 0.001.

Aβ immunohistochemistry from brain tissue sections was performed to investigate whether MAE supplementation altered Aβ deposition in *App^NL−G−F^* mice. As shown in Figure 6A, a significant reduction (*p* < 0.05) in Aβ staining was only observed in female MAE-treated *App^NL−G−F^* mice, compared to their vehicle group, while there was no significant change (*p* ≥ 0.05) in Aβ immunoreactivity in male mice. Aβ changes were further quantified by ELISA using hippocampal lysates from each animal (Figure 6B). In male *App^NL−G−F^* mice, MAE supplementation significantly reduced (*p* < 0.05) levels of soluble Aβ 1-40 and insoluble Aβ 1-42. In addition, female *App^NL−G−F^* mice supplemented with MAE showed a significant decrease (*p* < 0.05) in insoluble Aβ 1-40 levels.

### 3.9. Effects of MAE Supplementation on Microgliosis

**Figure 7:**
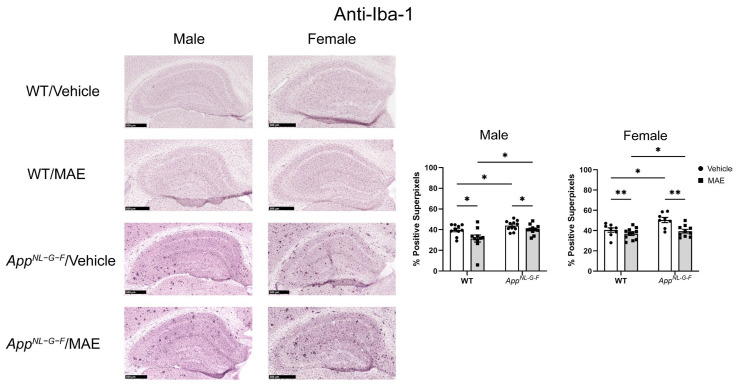
Effects of mushroom aqueous extract (MAE) supplementation on alteration of microgliosis in the hippocampus of wild-type (WT) and *App^NL−G−F^* mice after 8-week feeding. The brain sections of each animal were stained using anti-Iba-1 immunohistochemistry. Scale bar, 500 μm. The percentages of Iba-1-positive superpixels in the hippocampus were determined from at least three sections per mouse in each treatment group. Values are expressed as mean ± SEM (*n* = 8–11 per group). Two-way ANOVA followed by Tukey’s test was performed to determine statistical differences; * *p* < 0.05 and ** *p* < 0.01.

The activation of microglia is a commonly recognized neuroinflammatory process. Microglial reactivity was investigated to determine the impact of MAE supplementation by immunostaining for the ionized calcium-binding adapter molecule-1 (Iba-1) protein in brain sections. As shown in Figure 7, basal levels of Iba-1 staining were detected in all WT groups. Male *App^NL−G−F^* mice treated with vehicle had significantly higher (*p* < 0.05) levels of Iba-1 staining compared to vehicle-treated WT mice. A similar finding was also observed in MAE treatment groups, in which levels of the Iba-1 staining of male *App^NL−G−F^* mice were significantly higher (*p* < 0.05) than in male WT mice. Significant differences between genotypes were found in female mice. Comparing diet intervention, MAE supplementation significantly decreased (*p* < 0.05) the levels of Iba-1 staining in both WT and *App^NL−G−F^* groups in both sexes.

### 3.10. Effects of MAE Supplementation on Astrogliosis

**Figure 8:**
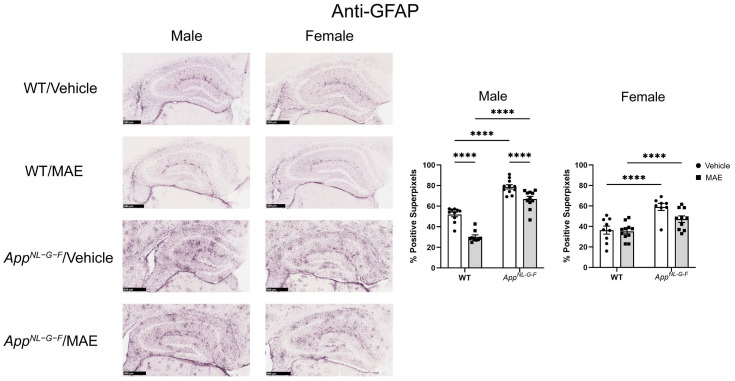
Effects of mushroom aqueous extract (MAE) supplementation on the alteration of astrogliosis in the hippocampus of wild-type (WT) and *App^NL−G−F^* mice after 8-week feeding. The brain sections of each animal were stained using an anti-GFAP antibody. Scale bar, 500 μm. The percentages of GFAP-positive superpixels in the hippocampus were determined from at least three sections per mouse in each treatment group. Values are expressed as mean ± SEM (*n* = 8–11 per group). Two-way ANOVA followed by Tukey’s test was performed to determine statistical differences; **** *p* < 0.0001.

Astrocytes are the most abundant glial cells in the brain and have an important role in neuroinflammatory regulation. To determine whether MAE supplementation changed astrogliosis, the brain sections were immunostained for the glial acidic fibrillary protein (GFAP), as shown in Figure 8. In both male and female WT groups, basal levels of GFAP staining were detected. Comparing genotypes, male and female *App^NL−G−F^* mice treated with vehicle had significantly higher (*p* < 0.05) levels of GFAP staining compared to vehicle-treated WT mice. Similarly, the GFAP staining levels of male and female MAE-supplemented *App^NL−G−F^* mice were significantly higher (*p* < 0.05) than those of MAE-fed WT groups. In addition, MAE supplementation significantly reduced (*p* < 0.05) levels of GFAP staining in male WT and *App^NL−G−F^* mice, while there was no significant change (*p* ≥ 0.05) of GFAP levels after MAE treatment in female WT and *App^NL−G−F^* mice.

### 3.11. Effects of MAE Supplementation on Working Memory

**Figure 9:**
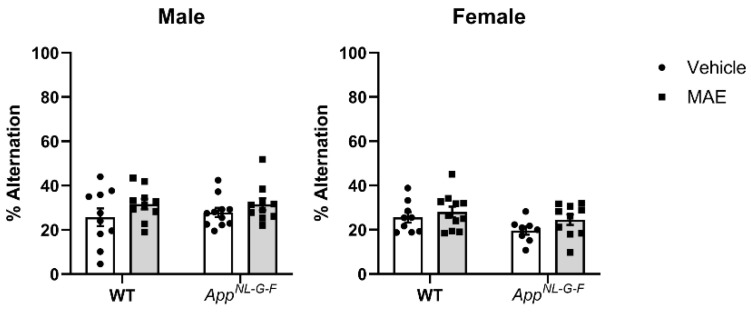
Effects of mushroom aqueous extract (MAE) supplementation on the working memory of wild-type (WT) and *App^NL−G−F^* mice. All mice were subjected to a cross-maze test to assess working memory function after 8 weeks of treatment. The percentage of alternations was calculated and shown as the mean ± SEM (*n* = 8–11 per group). Two-way ANOVA followed by Tukey’s test was performed to determine statistical differences.

Memory loss is a cognitive impairment associated with Alzheimer’s disease. Therefore, a cross-maze test was performed to evaluate whether MAE supplementation for 8 weeks affected short-term memory. Surprisingly, there was no significant difference (*p* ≥ 0.05) in % alternation observed in all treated groups, comparing between genotypes and diet treatment (Figure 9).

## 4. Discussion

The present study investigated the anti-inflammatory activity of a mushroom aqueous extract from *P. atrovolvatus* fruiting bodies. According to our previous studies, the bamboo mushroom *P. atrovolvatus* exerts potential benefits on gut health by promoting the growth of probiotic bacteria and SCFA production in correlation to its β-glucan content [29]. In this study, the MAE comprised 46.30% total glucans, a homopolysaccharide, as a major component. Therefore, the main subunit of the extract is D-glucose linked by α- and β-glycosidic bonds to form the structures α- and β-glucan, respectively [15]. The MAE contained 30.06% β-glucan, which is a well-documented dietary fiber with gut health benefits and immune-modulating activities [39]. In addition, *P. atrovolvatus* is known to be a source of essential amino acids, including valine, threonine, histidine, and phenylalanine, as well as a non-essential amino acid, alanine [40]. However, the structure characterization and determination of other components should be further elucidated. After 8 weeks of MAE treatment, the amount of consumed vehicle +/− MAE was in the range of 6–8 g/mouse/day. As a result, the MAE-received doses in the MAE-treated groups were higher than the expected 100 mg/kg BW/mouse/day. However, there was no effect on mouse body weight throughout the study.

Gut inflammation and barrier integrity were investigated to evaluate whether MAE supplementation had effects on the gut health of all animals. Gut inflammation was followed by the measurement of lipocalin levels in colons. Neutrophil gelatinase-associated lipocalin, also known as NGAL, lipocalin-2, or LCN2, is a biomarker for colonic inflammation. This 24 kDa glycoprotein is highly expressed in inflamed intestinal and colonic epithelial cells [41]. Our results showed that MAE did not promote lipocalin levels in the colons of all treatment groups. However, the lipocalin levels of female *App^NL−G−F^* mice were observed to be higher than WT mice in both vehicle- and MAE-treated groups. This result agreed with a previous study that demonstrated that *App^NL−G−F^* mice had significantly greater lipocalin levels than a WT group, possibly due to increased intestinal permeability and proinflammatory cytokines in *App^NL−G−F^* mice [38]. In addition, MAE supplementation did not affect gut barrier integrity since there were no significant changes in tight junction protein (ZO-1) levels in all mouse groups. Since MAE was prepared from a mushroom raw material grown from the soil, LPS might be a contaminant in the extract from the preparation and extraction process. However, LPS was removed by an alkaline–ethanol treatment before feeding to the mice. In addition, MAE supplementation did not increase LPS concentrations in the serum of any treatment groups, suggesting that no increase in gut permeability occurred with feeding. Therefore, the administration of MAE for 8 weeks had no adverse effects on gut health and did not induce an overall inflammatory response.

The comprehensive assessment of the immune response to the MAE in the *App^NL−G−F^* mice involved the evaluation of key immune markers across multiple tissue compartments. MAE administration modulated several innate immune markers, including increased expression of antibodies and phagocytic receptors in the myeloid cell populations. Specifically, splenocyte immune profiling revealed a significant increase in the frequency of IgM^+^ cells, indicating enhanced humoral adaptive immunity, as well as the increased frequency of IA_IE^+^- and CD14^+^-expressing cells, reflecting more pronounced innate immune activation in MAE-administered *App^NL−G−F^* female mice compared to their vehicle controls. In contrast, the analysis of specific T cell subsets, such as CD4^+^ and CD8^+^, did not mirror this clear sex-specific pattern of immune responses to MAE. Our data from blood revealed distinct MAE-induced changes in the T cell subsets, specifically in the *App^NL−G−F^* male mice. Notably, the levels of circulating CD8^+^ cytotoxic T cells were significantly reduced in the *App^NL−G−F^* male mice that received MAE compared to vehicle controls, suggestive of the sequestration or redistribution of these subsets out of the peripheral compartment. The sex-based differences were more pronounced when examining the cervical lymph nodes, a tissue compartment that is the primary drainage site for lymph and immune cells from the brain. *App^NL−G−F^* male mice that received MAE showed a significant increase in the frequency of cytotoxic CD8^+^ T cells within the cervical lymph nodes compared to their wild-type counterparts. Interestingly, MAE-administered wild-type males displayed a significant reduction in the frequency of cytotoxic T cells compared to their vehicle controls. Intriguingly, this pattern was reversed when examining the frequency of CD4^+^ T helper cell subsets. Taken together, the immunophenotyping findings from our study reveal a complex immunomodulatory response to MAE, particularly the T cell dynamics in the *App^NL−G−F^* male mice. Specifically, the decrease in circulating cytotoxic CD8^+^ T cells, coupled with the simultaneous increase in the cervical lymph nodes, indicates that the MAE may have induced preferential trafficking or homing of these cells to the lymph nodes draining the CNS while depleting them from the peripheral blood. To our knowledge, the observed MAE-induced compartment-specific and sex- and genotype-dependent changes in the immune repertoire have not been widely reported in the literature, highlighting the insights provided by our study. Importantly, our data demonstrate MAE-induced innate/antibody-driven responses seen in *App^NL−G−F^* females versus T-cell-mediated responses seen in *App^NL−G−F^* males, reflecting underlying disparities in the activation and recruitment of distinct immune pathways.

The immunomodulating activity of MAE might be due to the presence of β-glucan, which can stimulate innate immunity. Pattern recognition receptors (PRRs) such as dectin-1 and Toll-like receptors are expressed on the cell surface of innate immune cells. These PRRs recognize pathogens via binding to pathogen-associated molecular patterns such as β-glucan and LPS. The binding of β-glucan to dectin-1 on macrophages or dendritic cells activates adaptive immune cells such as B and T lymphocytes by secreting various cytokines such as IL-6 and TNF-α [42,43]. Our results agree with previous studies that demonstrated that mushrooms and their polysaccharides exhibit immune-boosting properties by the modulation of immune cells. Gue et al. reported that an oral solution of *P. indusiata* could repair immune function in radiation-damaged rats by promoting the proliferation of CD4^+^, CD16^+^, and CD57^+^ cells, as well as decreasing the CD8^+^ population [44]. Polysaccharides from *Pleurotus ostreatus* and *Dictyophora echinovolvata* increased the concentration of IgA, IgG, and IgM, as well as up-regulated splenic lymphocyte changes in immunosuppressed mice [45,46].

SCFAs are gut-microbial-derived metabolites that contribute various health benefits to the host and act as key molecules in the gut–brain crosstalk. After their production, SCFAs are absorbed by colonocytes and transported into the portal circulation, allowing SCFAs to cross the BBB into the brain [47,48]. In this study, we quantified SCFAs in serum and the brain to assess whether MAE supplementation altered their levels. Increased pyruvate levels were only observed in the serum of male MAE-supplemented WT and *App^NL−G−F^* mice, compared to their vehicle controls, with no changes observed in female mice. The increase of pyruvate might be a result of the fermentation of non-digestible carbohydrates, in which β-glucan and other polysaccharides in MAE were utilized by the gut microbiota and converted to SCFAs. In carbohydrate fermentation, the Embden–Meyerhof–Parnas pathway and the pentose–phosphate pathway are major metabolic pathways that first convert monosaccharides into phosphoenolpyruvate (PEP). Subsequently, PEP is changed into pyruvate, which is a substrate for the formation of SCFAs via different pathways [49]. There were no alterations of acetate, propionate, butyrate, isobutyrate, and lactate levels in either serum or brains of all mouse groups, indicating that MAE was unable to increase the majority of produced SCFAs, perhaps limiting the role of these molecules as explanations for changes in the brain.

Changes in cytokine levels in mouse brains were also evaluated. Male *App^NL−G−F^* mice had higher levels of IL-1β, which is known for its pro-inflammatory functions, compared to WT mice. This indicated that *App^NL−G−F^* mice were likely in an inflammatory state. However, there was no obvious upregulation of other proinflammatory cytokines in *App^NL−G−F^* compared to WT mice. After the intervention, MAE promoted levels of IL-22 in both genotype male groups. IL-22, a member of the IL-10 family, is involved in the proinflammatory response, tissue injury, and repair and is associated with neurodegenerative diseases [50]. Previous studies demonstrate the proinflammatory functions of IL-22 in the brain [51,52]. However, the anti-neuroinflammatory potential of IL-22 has also been reported, making the consequences of this selective upregulation unclear [53]. In female groups, MAE increased TNF-α, a potent pro-inflammatory cytokine, in WT mice but decreased it in *App^NL−G−F^* mice. The reason for this inverse effect in female mice based on genotype is unclear. Overall, MAE had no robust effect on the regulation of brain cytokines, one of the key mediators in neuroinflammatory responses.

One of the most common pathologies of AD is the extracellular accumulation of Aβ, which is hypothesized to lead to reactive glial cells and proinflammatory responses [11]. In this study, therefore, the immunohistochemistry of Aβ accumulation and gliosis was further investigated to determine neuroinflammatory responses in mouse brains. Our findings demonstrated that MAE altered Aβ accumulation in the hippocampus of male and female *App^NL−G−F^* mice. Although immunohistochemistry in hippocampi illustrated that MAE decreased Aβ accumulation in the female but not male *App^NL−G−F^* mice, the Aβ levels detected by ELISA were reduced by MAE intervention in both groups. This suggested that overall, there was a decrease in Aβ accumulation due to the MAE consumption. It is currently unclear if this is due to decreased production or increased clearance. Since previous studies have reported a neuroprotective effect of pyruvate in animal models, the inhibition of Aβ load might be related to the elevated serum pyruvate levels in *App^NL−G−F^* mice. The systemic administration of sodium pyruvate (500 mg/kg BW/mouse/day) through an intraperitoneal method for 10 days markedly suppressed neuronal death and reduced ROS levels in the hippocampus, resulting in an improvement of cognitive impairment in a rat model of AD [54]. Also, chronic treatment with pyruvate via intraperitoneal injection at a dose of 500 mg/kg BW/mouse/day for 9 months reduced oxidative stress in neurons and promoted beneficial cognitive effects on short and long-term memory deficits in 3xTg-AD mice [55].

Although the mechanism underlying the reduction in Aβ accumulation in MAE-supplemented groups is still unclear, our results are in agreement with previous studies indicating that mushroom polysaccharides can reduce the Aβ burden in AD animal models. The administration of polysaccharides from *G. frondosa* (10 mg/kg BW/mouse/day) for 3 months, from *Pleurotus ostreatus* (400 mg/kg BW/mouse/day) for 8 weeks, and from *Armillaria mellea* (100 mg/kg BW/mouse/day) for 4 weeks reduced Aβ accumulation in APP/PS1 and AlCl_3_ and D-galactose-induced mouse models of AD [56,57,58]. According to these previous reports, the mechanisms involved with Aβ clearance included the enhancement of microglial recruitment around Aβ plaques, promoting Aβ phagocytosis by microglial, regulating nuclear factor-E2-related factor 2 (Nrf2) signaling, and inhibiting NF-κB activation, along with the suppression of oxidative stress and phosphorylated tau aggregation. Future work using the MAE will need to focus on altered microglial clearance of Aβ.

In the CNS, glial cells include astrocytes, microglia, and oligodendrocytes, with astrocytes being the most abundant [59]. Under AD conditions, the microglia and astrocytes are activated and hypothesized to release proinflammatory mediators. Indeed, several previous studies have reported a correlation between the presence of Aβ and the activation of microglia and astrocytes [8,60,61]. In the current study, microgliosis and astrogliosis were investigated via immunostaining for Iba-1 and GFAP, respectively. As expected, male and female *App^NL−G−F^* mice showed higher levels of Iba-1 staining when compared to their WT counterparts. Both male and female *App^NL−G−F^* mice also had more robust immunoreactivity of GFAP compared to their WT control mice. This indicated that overall gliosis was exacerbated in the *App^NL−G−F^* mouse model. After 8 weeks of MAE feeding, MAE reduced levels of Iba-1 immunoreactivity in all treatment groups, indicating that MAE suppresses microgliosis in the hippocampus of both WT and *App^NL−G−F^* mice, regardless of sex or diet. However, the MAE supplementation suppressed astrogliosis in only male but not female WT and *App^NL−G−F^* mice. The lack of ability for MAE to attenuate astrogliosis in female mice could indicate a preferential response on microglia or, more likely, a need to better characterize astrocyte phenotype beyond GFAP immunoreactivity in future work. Our findings are broadly consistent with a previous study reporting that mushroom polysaccharides can alleviate gliosis in an AD mouse model. The oral administration of polysaccharides from *Boletus aereus* Bull. mushroom (50 and 100 mg/kg BW/day) for 8 weeks inhibited the activation of microglia demonstrated by reduced Iba-1 staining, decreased astrogliosis evidenced by lower GFAP levels, and suppressed production of proinflammatory cytokines, IL-1β and IL-6, in APP/PS1 mice [62].

Working memory assessed by the cross-maze test showed that MAE supplementation did not affect memory performance in the mouse groups. Also, there was no significant difference in % alternation between WT and *App^NL−G−F^* mice. It is possible that the test selected was not sensitive enough to detect memory differences across genotypes. Alternatively, memory deficits in the *App^NL−G−F^* line may not have yet appeared by the age of our testing. Other behavioral tests involving spatial and learning memory, as well as anxiety, should be further investigated along with an older cohort of mice in future work.

Overall, MAE from *P. atrovolvatus* exerted the ability to alter Aβ levels and reactive gliosis in an AD mouse model, possibly through the gut–brain axis. The WT and *App^NL−G−F^* mice received MAE containing approximately 30% β-glucan, which might have been utilized by the colon microbiota to ultimately increase pyruvate production through anerobic bacterial fermentation. The produced pyruvate could have been absorbed by the colonocytes and transported into the circulation to explain the increase observed in serum. The neuroprotective effects of pyruvate, along with the altered immune profile in cervical lymph nodes, could have contributed to the reduced Aβ accumulation and the resulting changes in microgliosis and astrogliosis in the brain.

## 5. Conclusions

Mushroom aqueous extract from the *P. atrovolvatus* fruiting body contained 30% β-glucan. After intervention, the mushroom extract had no adverse effects on gut inflammation and gut barrier integrity and did not induce overall inflammatory changes. Supplementation with mushroom extract enhanced immune responses in the spleens of female *App^NL−G−F^* mice via the upregulation of B cell-humoral response and the engagement of MHC class II and monocytes–macrophages. Also, MAE intervention exerted an immunomodulatory effect in the draining cervical lymph nodes of male wild-type mice by increasing helper T cells and reducing cytotoxic T cell populations. In addition, the production of pyruvate was promoted in both wild-type and *App^NL−G−F^* mice, possibly due to the fermentation of mushroom extract by gut microbiota. The mushroom extract reduced Aβ deposition, microgliosis, and astrogliosis in the hippocampus most robustly in male *App^NL−G−F^* mice. Our findings suggest that the *P. atrovolvatus* mushroom has the neuroprotective potential to alleviate gliosis and Aβ changes in Alzheimer’s disease mouse models, although the mechanism is unclear and may be unrelated to brain changes in short-chain fatty acid levels or immune cells. The protein–polysaccharide complex structure of MAE, the administration method, and the limited treatment period are limitations of this study. Future work is needed to determine whether there is a key bioactive molecule, deliver more precise and prolonged dosing, assess microbiome changes, and more extensively evaluate any cognitive benefits. In particular, further investigation of gut microbiome profiling and determinations of neurotransmitter, bile acid, and gut hormone changes should be conducted, along with assessing other molecular parameters in the brain, to expand our understanding of the mechanisms of action.

## Figures and Tables

**Table 1:** Consumption of MediGel^®^Sucralose and calculated dose of MAE.

Treatment Group	MediGel^®^Sucralose (g/Mouse/day)	Received Dose (mg/kg BW/Mouse/day)	
		MAE	β-Glucan
Male/WT/vehicle	7.22 ± 0.06	-	-
Male/WT/MAE	7.92 ± 0.08	132.0 ± 1.3	39.61 ± 0.40
Male/*App^NL−G−F^*/vehicle	7.27 ± 0.07	-	-
Male/*App^NL−G−F^*/MAE	8.17 ± 0.10	138.9 ± 1.7	41.76 ± 0.52
Female/WT/vehicle	7.43 ± 0.09	-	-
Female/WT/MAE	7.05 ± 0.07	143.1 ± 1.4	42.93 ± 0.44
Female/*App^NL−G−F^*/vehicle	6.71 ± 0.06	-	-
Female/*App^NL−G−F^*/MAE	6.85 ± 0.08	139.3 ± 1.6	41.78 ± 0.47

Values are expressed as mean ± SEM.

## Data Availability

The original contributions presented in this study are included in the article. Further inquiries can be directed to the corresponding author.
